# Role of salvianolic acid B in the treatment of acute ischemic stroke: a systematic review and meta-analysis of animal models

**DOI:** 10.3389/fphar.2024.1479765

**Published:** 2024-12-24

**Authors:** Jiashan Wang, Pingping Su, Chenyu Wan, Yingqi Xu, Junyue Huang, Jianli Niu, Zhuqing Jin

**Affiliations:** ^1^ The Third School of Clinical Medicine, Zhejiang Chinese Medical University, Hangzhou, China; ^2^ School of Clinical Medicine, The Affiliated Hospital of Hangzhou Normal University, Hangzhou, Zhejiang, China; ^3^ The Second School of Clinical Medicine, Zhejiang Chinese Medical University, Hangzhou, China; ^4^ Office of Human Research, Memorial Healthcare System, Florida, United States; ^5^ School of Basic Medical Sciences, Zhejiang Chinese Medical University, Hangzhou, China

**Keywords:** salvianolic acid B, acute ischemic stroke injury, MCAO, Neuroprotection, meta-analysis

## Abstract

**Background:**

Salvianolic acid B (Sal B) is potentially the most valuable water-soluble active component in Salvia miltiorrhiza. Its chemical formula contains multiple phenolic hydroxyl groups, so it has a strong antioxidant capacity.

**Objective:**

We aim to investigate the efficacy and the potential mechanism of Sal B in the treatment of acute ischemic stroke injury.

**Materials and methods:**

CNKI, VIP, WanFang, SinoMed, PubMed and Web of Science were searched for all the literature related to Sal B in the treatment of acute ischemic stroke before August 2024. The methodological quality was assessed using an inspection scale combining the CAMARADES checklist and the new STAIR criteria. Data were analyzed using RevMan5.4 software.

**Results:**

A total of 14 articles were included. Sal B could effectively reduce infarct size, neurological deficit score, brain edema index, and brain water content in cerebral ischemic animals. Sal B could not only increase the content of superoxide dismutase (SOD) and decrease the content of malondialdehyde (MDA) to achieve anti-oxidative stress, but also reduce the level of interleukin-1β (IL-1β) protein to achieve anti-inflammatory response, and reduce the number of TUNEL cells to reflect its anti-apoptosis effect. In addition, Sal B can improve energy metabolism by increasing the content of energy charge (EC) and phosphocreatine (PCr), and maintaining ion balance via Na^+^/K^+^ ATPase activity, resulting in the neuroprotective effects against acute ischemic stroke injury.

**Conclusion:**

This study showed that Sal B could significantly protect against acute ischemic stroke injury, mainly through anti-oxidative stress, anti-inflammatory response, anti-apoptosis, improving energy metabolism, and stabilizing ion balance.

## 1 Introduction

*Salvia miltiorrhiza* is the dried root and rhizome of *Salvia miltiorrhiza* in the Labiatae family, which has the effects of vasodilation, myocardial protection, anti-atherosclerosis and anti-thrombosis (Giugliano et al., 2020; Wang G. et al., 2022; He and Chen, 2020; Rosińska et al., 2017), and is widely used in clinical treatment of various cardiovascular and cerebrovascular diseases, including coronary heart disease, myocardial infarction and stroke caused by cerebral ischemia, dementia (Fang et al., 2018; Qin et al., 2021; Guo and Wang, 2022). Chinese Pharmacopoeia (2020 edition) stipulated that the total amount of tanshinone-containing tanshinone I, IIA and cryptotanshinone should not be less than 0.25%, and salvianolic acid B (Sal B) should not be less than 3.0% as the quality evaluation standard of salvianolic acid. Sal B is a water-soluble compound, while tanshinone I, tanshinone IIA and cryptotanshinone are lipid-soluble compounds (Chinese Pharmacopoeia Commission, 2020). Therefore, Sal B is potentially the most valuable water-soluble active component in *Salvia miltiorrhiza*.

**FIGURE 1:**
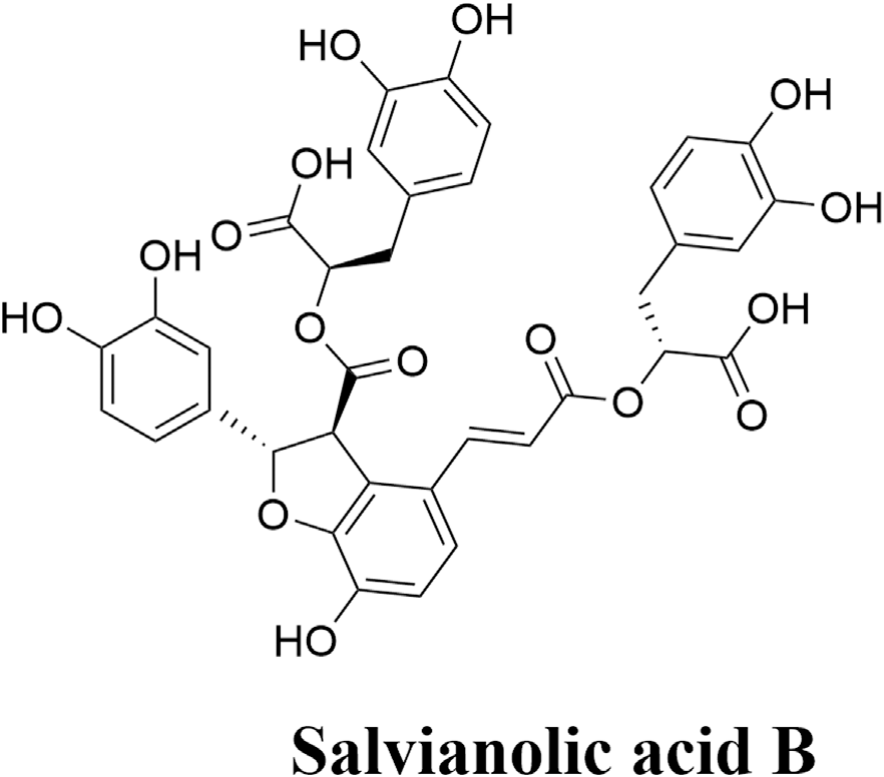
Salvianolic acid B chemical formula.

Sal B is a monomer compound formed by the condensation of three molecules of 3,4-dihydroxyphenyllactic acid and one molecule of caffeic acid (Figure 1) (He et al., 2023). There are several phenolic hydroxyl groups in the molecular structure of Sal B, indicating strong antioxidant activity property (Loaiza-Cano et al., 2020). Recently, research results show that Sal B possesses various pharmacological characteristics, such as anti-inflammatory and anti-apoptotic effects contributing to improve the injury of acute ischemic stroke (Lee et al., 2023; Xiao et al., 2020; Zheng et al., 2021). Sal B has minor side effects with a higher margin of safety (Ding et al., 2017).

Several studies have reported the neuroprotective effects of Sal B on acute ischemic stroke injury (Zhu et al., 2022). The mechanisms underlying its neuroprotective effects have been investigated in animal models of acute ischemic stroke in recent years. In this study, we conducted a systematical review and meta-analysis of studies in animals, aiming at evaluating the therapeutic effects of Sal B against acute ischemic stroke and determining the relevant mechanisms of Sal B in the treatment of acute ischemic stroke, hopefully providing scientific evidence for the clinical application of Sal B in protecting against acute ischemic stroke injury.

## 2 Materials and methods

This study was reported in accordance with the PRISMA principles (Moher et al., 2009) and the Cochrane Collaboration guidance (Cumpston et al., 2019).

### 2.1 Literature retrieval strategy

In this study, the Chinese Medical Subject Headings (MeSH) of the National Library of Medicine was used to determine the item term of “ischemic stroke” (DeMars and Perruso, 2022), and the following search strategies were established according to the results: (Sal B) AND (ischemic stroke OR cerebral ischemia OR cerebrovascular disease OR cerebral infarction OR cerebral infarction OR cerebral embolism). Six databases including CNKI, VIP, WanFang, SinoMed, PubMed and Web of Science were searched by two researchers independently. The search period was up to August 2024, and the language of the publications was not restricted. The search results were imported into reference management software.

### 2.2 Study inclusion and exclusion criteria

Experimental studies in animal models of acute ischemic stroke were included, with no restrictions on animal species, strain, sex, or modeling methods. The experimental group was the acute ischemic stroke model injected with Sal B, and the control group was the acute ischemic stroke model without Sal B injection.

Exclusion criteria were as follows: (1) Duplicate or irrelevant literature; (2) Reviews, conference proceedings, dissertations, informal journals, catalogs, etc.; (3) Clinical and other *in vitro* studies; (4) Animal models unrelated to acute ischemic stroke; (5) Studies involving combination therapies; (6) Studies with incomplete or missing data; (7) Outcome measures could not be extracted or combined.

### 2.3 Outcome measures

All indicators were divided into primary indicators and secondary indicators according to whether they could directly reflect the efficacy (Li and Zhang, 2021; Zhang et al., 2005). Primary outcome measures included infarct size, neurological deficit score, and brain edema index (brain index, brain water content). Secondary indicators included oxidative stress markers (SOD, MDA), inflammatory markers (IL-1β protein level), apoptosis index (number of TUNEL-positive cells), and cytotoxic edema markers (EC, PCr, Na^+^/K^+^ ATPase activity, lactate).

### 2.4 Literature screening and data extraction

Two researchers searched independently according to strict inclusion and exclusion criteria, and all data were screened and extracted in Noteexpress software. Any disagreements on the eligibility of studies were resolved by discussing with the third investigator.

Data were extracted from original text, tables, figures, supplementary materials and methods, and references. GetData Graph Digitizer software was used to extract data in literature that only presented data in the form of images. The experimental part included intervention design, administration route, administration dose, treatment time, and outcome indicators, in which the outcome indicator was the difference of Sal B before and after intervention. The model part included animal species, strain, sex, age, weight, ischemia type and modeling method. The material section included drug purity, source, etc. In addition, general information such as author, country, and year of publication was collected.

### 2.5 Quality assessment and risk of bias

An inspection scale (Aliena-Valero et al., 2021) including the CAMARADES (Sena et al., 2007) criteria and the STAIR criteria (Fisher et al., 2009) was used for quality assessment: (1) Published in a peer-reviewed journal; (2) Temperature control declaration; (3) Randomization; (4) Hidden allocation; (5) Blinded evaluation; (6) Use of anesthetics without neuroprotective properties (e.g., chloral hydrate); (7) In addition to ischemia, the experimental animals had complications; (8) Clarifying the sample size; (9) Comply with animal welfare regulations; (10) Reported potential conflicts of interest; (11) Timely detection of animal physiological indicators; (12) Clear inclusion and exclusion criteria; (13) There were reports of animal culling; (14) Funding report (15) Modeling success was confirmed by techniques such as laser Doppler or perfusion imaging.

### 2.6 Statistical analysis

Statistical software Rev man 5.4 was used to analyze the collected data. The mean difference (MD) or standardized mean difference (SMD) was used to report the pooled effect estimate for continuous variables with their 95% confidence interval (CI). Heterogeneity among studies was categorized by the I^2^ statistic as follows: I^2^<50% indicated low or non-significant heterogeneity, and the fixed effect models were used, and I^2^ > 50%, represented high heterogeneity, and the random effect models were used. For the literature with high heterogeneity, subgroup analysis or sensitivity analysis was further analyzed according to the actual situation to determine the source of heterogeneity.

## 3 Result

### 3.1 Included literature

**FIGURE 2:**
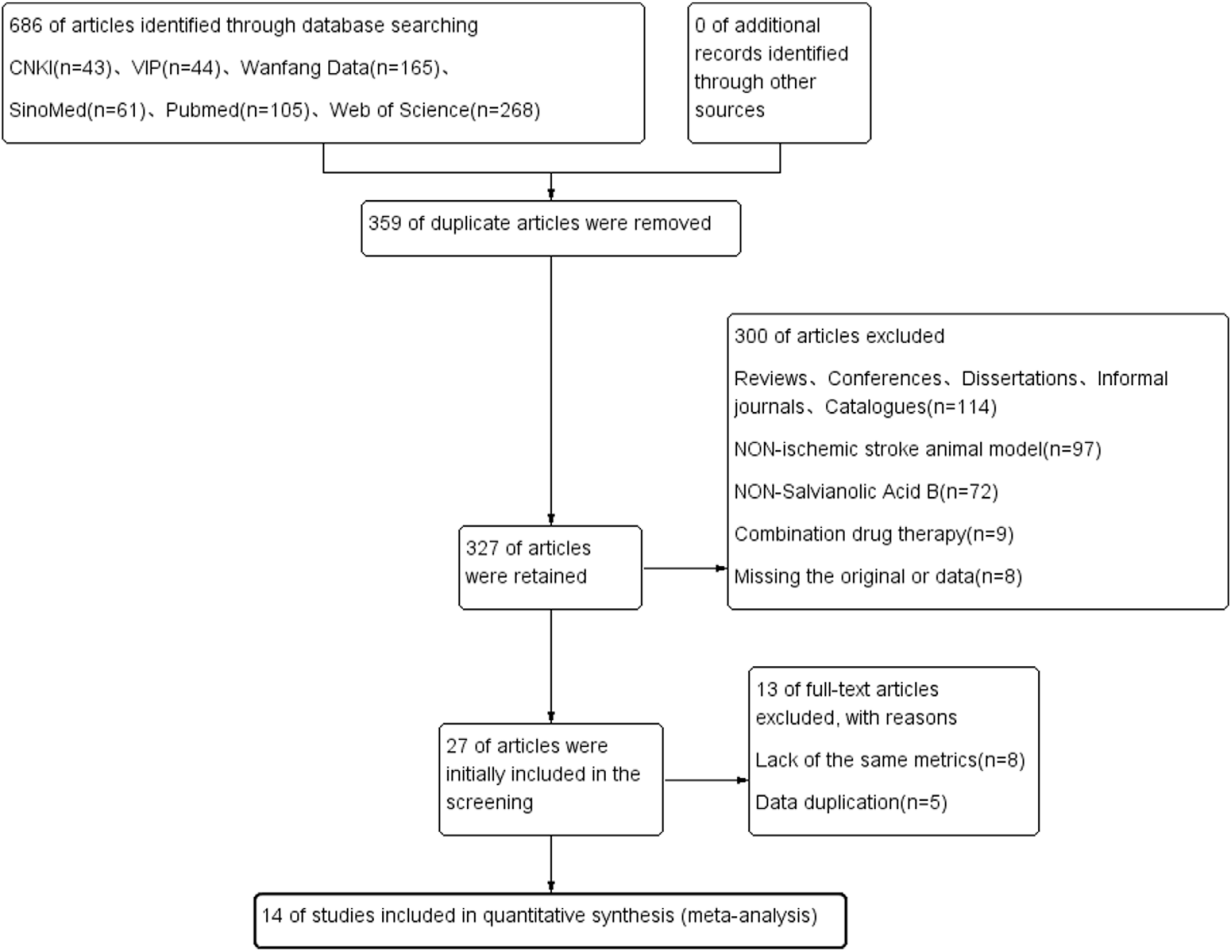
Literature search flow chart.

A total of 686 literatures were retrieved from six databases including CNKI and Wanfang. Among these, 359 duplicates and 114 reviews, conferences, dissertations, unofficial journals, and catalog literature were excluded. We screened the literature according to the title and abstract of the literature, and excluded the literature of non-non-animal models (n = 97), non-salvianolic acid B (n = 72), combination drugs (n = 9), and loss of original text and incomplete data reporting (n = 9), leaving 27 literatures were reviewed, and a further 13 excluded. The remaining 14 articles finally met the inclusion criteria and were included in the meta-analysis. The study selection flow chart is shown in Figure 2.

### 3.2 Study characteristics and quality assessment

Tables 1, 2 summarize the characteristics of the included studies. Of the 14 studies, 7 studies were conducted in rats and 7 in mice. The dosage of Sal B ranged from 10 mg/kg to 192 mg/kg, and was administrated via the tail veins or intraperitoneal injections. All studies used anesthetics with no apparent intrinsic neuroprotective properties. Two studies did not explicitly mention the non-randomized controls, and the rest of the studies were randomized controlled trials. In the study design, two studies mentioned the use of a blinded method to assess outcomes, three studies had pre-specified inclusion and exclusion criteria, and one study reported the exclusion of animals for analysis. One study did not mention temperature control during ischemia induction. In addition, nine studies explicitly issued statements of compliance with animal welfare requirements, and eleven studies explicitly reported research funding. The results are shown in Table 3.

**TABLE 1:** Characteristics of the studies included.

Study	Country	Animal characteristics	Model	Modeling methods	Method of administration	Dose of Sal B	Time of administration	E	C	Outcome measures
Bi et al. (2022)	China	Male SD rats (200–230 g)	Ischemic stroke-reperfusion	Wire bolt method	Intraperitoneal injection	20 mg/kg	21 days	Sal B	Vehicle	①⑧
Chen et al. (2000)	China	Male Wistar rats (250–300 g)	Ischemic stroke-reperfusion	Wire bolt method	intravenous injection	10 mg/kg	25 h 10 min	Sal B	Vehicle	⑤⑥⑫
Fan et al. (2018)	China	Male C57BL/6 mice (20–25 g)	Ischemic stroke-reperfusion	Wire bolt method	Intraperitoneal injection	60 mg/kg	25 h	Sal B	Vehicle	①⑦
Fang et al. (2016)	China	Male NIH mice (18–22 g)	Ischemic stroke-reperfusion	Vascular occlusion	Tail vein injection	22.5 mg/kg	12 h 30 min	Sal B	Vehicle	③④
Jiang et al. (2007)	China	Male NIH mice (28–32 g)	Permanent ischemic stroke	Wire bolt method	Tail vein injection	22.5 mg/kg	1 h	Sal B	Vehicle	③④⑨⑩⑪⑫
Lv et al. (2019)	China	SD rats	Ischemic stroke-reperfusion	Wire bolt method	Intraperitoneal injection	50 mg/kg	4 days 2 h	Sal B	Vehicle	⑤⑥⑧
Wang G. et al. (2019)	China	Male SD rats (280 ± 10 g)	Ischemic stroke-reperfusion	Wire bolt method	Intraperitoneal injection	12 mg/kg	24 h	Sal B	Vehicle	①②③④⑤⑥
Wang H. et al. (2016)	China	Male ICR mice (25 ± 2 g)	Permanent ischemic stroke	Wire bolt method	Tail vein injection	22.5 mg/kg	6 h	Sal B	Vehicle	②
Wang X. et al. (2019)	China	Male SD rats (160–200 g)	Permanent ischemic stroke	Electrocoagulation model	Tail vein injection	40 mg/kg	24 h 10 min	Sal B	Vehicle	①
Xiao et al. (2014)	China	Male ICR mice (23–25 g)	Permanent ischemic stroke	Wire bolt method	Tail vein injection	45 mg/kg	6 h	Sal B	Vehicle	①②③④
Zhang et al. (2007)	China	Male NIH mice (28–32 g)	Permanent ischemic stroke	Wire bolt method	Tail vein injection	22.5 mg/kg	1 h	Sal B	Vehicle	③④⑨⑩⑪
Zhao et al. (2008a)	China	Male Wistar rats (220–250 g)	Ischemic stroke-reperfusion	Wire bolt method	Intragastric administration	96 mg/kg	8 days 3 h	Sal B	Vehicle	②⑤⑥
Zhao et al. (2008b)	China	Male Wistar rats (220–250 g)	Ischemic stroke-reperfusion	Wire bolt method	Intragastric administration	192 mg/kg	8 days 3 h	Sal B	Vehicle	①⑧
Zheng et al. (2023)	China	Male CD1 mice (25–30 g)	Ischemic stroke-reperfusion	Wire bolt method	Intraperitoneal injection	30 mg/kg	73 h	Sal B	Vehicle	①④⑦

Abbreviations: SD, sprague dawley; Sal B, Salvianolic acid B; E, experimental group; C, control group.

Notes:① Infarct size; ② Neurological deficit score; ③ Brain index; ④ Brain water content; ⑤ MDA; ⑥ SOD; ⑦ IL-1β protein levels; ⑧ The number of TUNEL-positive cells; ⑨ EC; ⑩ PCr; ⑪ Na^+^/K^+^ ATPase, activity; ⑫ Lactate.

**TABLE 2:** Summary of Sal B used in the included studies.

Study	Cpound, concentration	Source	Purity	Quality control reported
Bi et al. (2022)	NR	NR	NR	NR
Chen et al. (2000)	Pure substance	The Institute of Medicinal Plant Development	>99%	HPLC
Fan et al. (2018)	Pure substance	Nanjing Spring and Autumn Biological Engineering Co., Ltd.	= 98%	HPLC
Fang et al. (2016)	Pure substance	National Institutes for Food and Drug Control	>98%	HPLC
Jiang et al. (2007)	Pure substance	National Institutes for Food and Drug Control	>98%	HPLC
Lv et al. (2019)	Pure substance	Shanghai yuanye Bio-Technology Co., Ltd.	≥98%	HPLC
Wang G. et al. (2019)	Pure substance	Shanghai yuanye Bio-Technology Co., Ltd.	≥98%	HPLC
Wang H. et al. (2016)	Pure substance	Chengdu Purefa Science and Technology Development Co., LTD.	= 98%	HPLC
Wang X. et al. (2019)	Pure substance	Sinopharm Group Guangdong Medi-World Pharmaceutical Co., Ltd.	≥98%	HPLC
Xiao et al. (2014)	Pure substance	Chengdu Purefa Science and Technology Development Co., LTD.	= 98%	HPLC
Zhang et al. (2007)	Pure substance	National Institutes for Food and Drug Control	>98%	HPLC
Zhao et al. (2008a)	Pure substance	TIANJIN ZHONGYI PHARMACEUTICAL CO., LTD.	≥98.5%	HPLC
Zhao et al. (2008b)	Pure substance	TIANJIN ZHONGYI PHARMACEUTICAL CO., LTD.	≥98.5%	HPLC
Zheng et al. (2023)	Pure substance	Beijing Hengyuan Qitian Chemical Technology Research Institute	= 99.02%	HPLC

Abbreviations: Sal B: Salvianolic acid B; NR: no report; HPLC: high performance liquid chromatography.

**TABLE 3:** Quality of literature evaluation.

Author	Country	1	2	3	4	5	6	7	8	9	10	QS (0–10)	11	12	13	14	15	QS (0–15)
Bi et al. (2022)	China		+	+		+	+		+	+	+	7				+		8
Chen et al. (2000)	China						+		+			2						2
Fan et al. (2018)	China		+	+			+		+	+	+	6				+	+	8
Fang et al. (2016)	China		+	+			+		+	+		5						5
Jiang et al. (2007)	China		+	+			+		+			4				+		5
Lv et al. (2019)	China		+				+		+			3		+		+		5
Wang G. et al. (2019)	China		+	+			+		+			4	+			+		6
Wang H. et al. (2016)	China		+	+			+		+	+		5				+		6
Wang X. et al. (2019)	China		+	+			+		+	+		5				+		6
Xiao et al. (2014)	China		+	+			+		+	+		5				+	+	7
Zhang et al. (2007)	China		+	+			+		+			4				+		5
Zhao et al. (2008a)	China		+	+			+		+	+		5		+		+		7
Zhao et al. (2008b)	China		+	+			+		+	+		5		+	+			7
Zheng et al. (2023)	China		+	+		+	+		+	+		6				+		7

Notes: (1) Published in a peer-reviewed journal; (2) Temperature control declaration; (3) Randomization; (4) Hidden allocation; (5) Blinded evaluation; (6) Use of anesthetics without neuroprotective properties (e.g., chloral hydrate); (7) In addition to ischemia, the experimental animals had complications; (8) Clarifying the sample size; (9) Comply with animal welfare regulations; (10) Reported potential conflicts of interest; (11) Timely detection of animal physiological indicators; (12) Clear inclusion and exclusion criteria; (13) There were reports of animal culling; (14) Funding report (15) Modeling success was confirmed by techniques such as laser Doppler or perfusion imaging.

### 3.3 Included literature

#### 3.3.1 Infarct size

Seven studies (Bi et al., 2022; Fan et al., 2018; Wang G. et al., 2019; Wang X. et al., 2019; Xiao et al., 2014; Zhao et al., 2008b; Zheng et al., 2023) reported the effects of Sal B on infarct size following acute ischemic stroke injury. The results showed that Sal B could effectively reduce infarct size (MD = −16.93, 95%CI = [−23.01, −10.85]; I^2^ = 97%, *P* < 0.0001) (Table 4).

**TABLE 4:** Meta-analysis results of each outcome indicator.

Outcome indicator	Heterogeneity test results		Effect models	Meta-analysis results		
	I^2^ (%)	*P*		Effect sizes	95%CI	*P*
Infarct size	97	<0.00001	Random	MD = −16.93	[−23.01, −10.85]	<0.0001
Neurological deficit score	75	0.008	Random	MD = −0.86	[−1.29, −0.42]	0.001
Brain index	91	<0.00001	Random	MD = −0.01	[−0.02–0.00]	0.02
Brain water content	99	<0.00001	Random	MD = −1.87	[−3.50, −0.23]	0.03
SOD	98	<0.00001	Random	MD = 13.06	[−3.26, 22.86]	0.0009
MDA	98	<0.00001	Random	MD = 4.90	[−7.51, 2.29]	0.0002
IL-1β protein level	83	0.01	Random	MD = 0.18	[−3.02, −0.03]	0.02
TUNEL-positive cells	97	<0.00001	Random	MD = −19.64	[−35.81, −3.47]	0.02
EC	10	0.29	Fixed	SMD = 0.05	[0.02, 0.08]	0.0004
PCr	0	0.50	Fixed	SMD = 10.49	[4.75, 16.22]	0.0003
Na^+^/K^+^ ATPase activity	0	0.97	Fixed	SMD = 0.59	[0.32, 0.86]	<0.00001
Lactate	88	0.04	Random	MD = −1.6	[−2.79, −0.40]	0.009

#### 3.3.2 Neurological deficit score

Four studies reported the effects of Sal B on neurological deficit score (Wang G. et al., 2019; Wang H. et al., 2016; Xiao et al., 2014; Zhao et al., 2008a). The results showed that Sal B could effectively reduce neurological deficit score (MD = −0.86, 95% CI = [−1.29, −0.42]; I^2^ = 75% *P* = 0.001) (Table 4).

#### 3.3.3 Brain index

Five studies reported the effects of Sal B on brain index following acute ischemic stroke injury (Fang et al., 2016; Jiang et al., 2007; Wang G. et al., 2019; Xiao et al., 2014; Zhang et al., 2007). The results showed that Sal B could effectively reduce the brain index (MD = −0.01, 95%CI = [-0.02, −0.00]; I^2^ = 91%; *p* = 0.02) (Table 4).

#### 3.3.4 Brain water content

Six studies (Fang et al., 2016; Jiang et al., 2007; Wang G. et al., 2019; Xiao et al., 2014; Zhang et al., 2007; Zheng et al., 2023) reported the effects of Sal B on brain water content following acute ischemic stroke injury. The results showed that Sal B could effectively reduce brain water content (MD = −1.87, 95%CI = [−3.50, −0.23]; I^2^ = 99%; *P* = 0.03) (Table 4).

#### 3.3.5 SOD

Four studies (Chen et al., 2000; Lv et al., 2019; Wang G. et al., 2019; Zhao et al., 2008a) reported the effects of Sal B on brain SOD content following acute ischemic stroke injury. The results showed that Sal B could significantly increase the activity of SOD (MD = 13.06, 95%CI = [−3.26, 22.86]; I^2^ = 98%; *P* = 0.0009) (Table 4).

#### 3.3.6 MDA

Four studies (Chen et al., 2000; Lv et al., 2019; Wang G. et al., 2019; Zhao et al., 2008a) reported the effects of Sal B on brain MDA content following acute ischemic stroke injury. The results showed that Sal B could significantly increase the activity of MDA (MD = 4.90, 95%CI = [−7.51, 2.29]; I^2^ = 98%; *P* = 0.0002) (Table 4).

#### 3.3.7 IL-1β protein levels

Two studies reported the effects of Sal B on IL-1β protein levels following cerebral ischemic injury (Fan et al., 2018; Zheng et al., 2023). The results showed that Sal B could effectively increase the content of IL-1β protein (MD = 0.18, 95%CI = [−3.02, −0.03]; I^2^ = 83%; *P* = 0.02) (Table 4).

#### 3.3.8 The number of TUNEL-positive cells

Three articles [24, 29, 36] reported the effects of Sal B on the number of TUNEL-positive cells in the ischemic brain tissues. The results showed that Sal B could effectively reduce the number of TUNEL-positive cells (MD = −19.64, 95%CI = [−35.81, −3.47]; I^2^ = 97%; *P* = 0.02) (Table 4).

#### 3.3.9 Energy charge

Two articles (Jiang et al., 2007; Zhang et al., 2007) reported the effects of Sal B on EC. The results showed that Sal B could effectively improve EC (SMD = 0.05, 95%CI = [0.02, 0.08]; I^2^ = 10%; *P* = 0.0004) (Table 4).

#### 3.3.10 Phosphocreatine

Two articles (Jiang et al., 2007; Zhang et al., 2007) reported the effects of Sal B on brain PCr activity. The results showed that Sal B could effectively improve the PCr content (SMD = 10.49, 95%CI = [4.75, 16.22]; I^2^ = 0%; *P* = 0.0003) (Table 4).

#### 3.3.11 Na^+^/K^+^ ATPase activity

Two studies reported the effects of Sal B on brain Na^+^/K^+^ ATPase activity (Jiang et al., 2007; Zhang et al., 2007). The results showed that Sal B could effectively improve Na^+^/K^+^ ATPase activity (SMD = 0.59, 95%CI = [0.32, 0.86]; I^2^ = 0%; *P* < 0.00001) (Table 4).

#### 3.3.12 Lactate

Two articles reported the effects of Sal B on brain lactate content (Chen et al., 2000; Jiang et al., 2007). The results showed that Sal B could effectively reduce brain lactate content (MD = −1.6, 95%CI = [−2.79, −0.40]; I^2^ = 88%; *P* = 0.009) (Table 4).

### 3.4 Sensitivity analysis

#### 3.4.1 Infarct size

**FIGURE 3:**
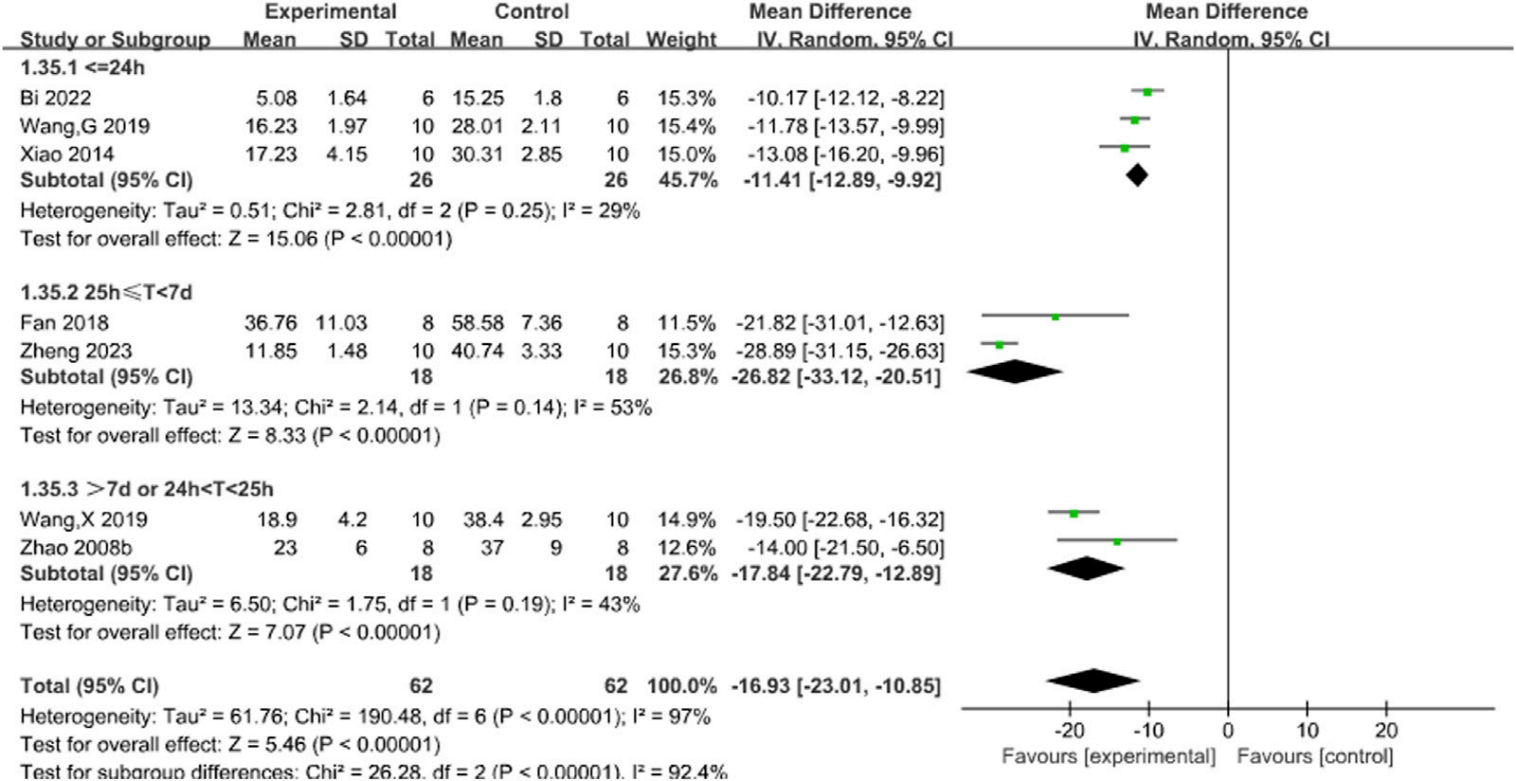
Forest chart of infarct size divided into subgroups according to the treatment time.

Subgroup analyses used the duration of treatment as the grouping criterion. We found that the I^2^ decreased to 29% for treatment duration of less than 24 h (*P* < 0.00001), 53% for treatment duration of 25 h to 7 days (*P* < 0.00001), and 43% for treatment duration of more than 7 days or more than 24 h or less than 25 h, suggesting that differences in treatment duration may be the main source of heterogeneity. The new pooled results showed that Sal B was effective in reducing infarct size (Figure 3).

#### 3.4.2 Neurological deficit score

**FIGURE 4:**
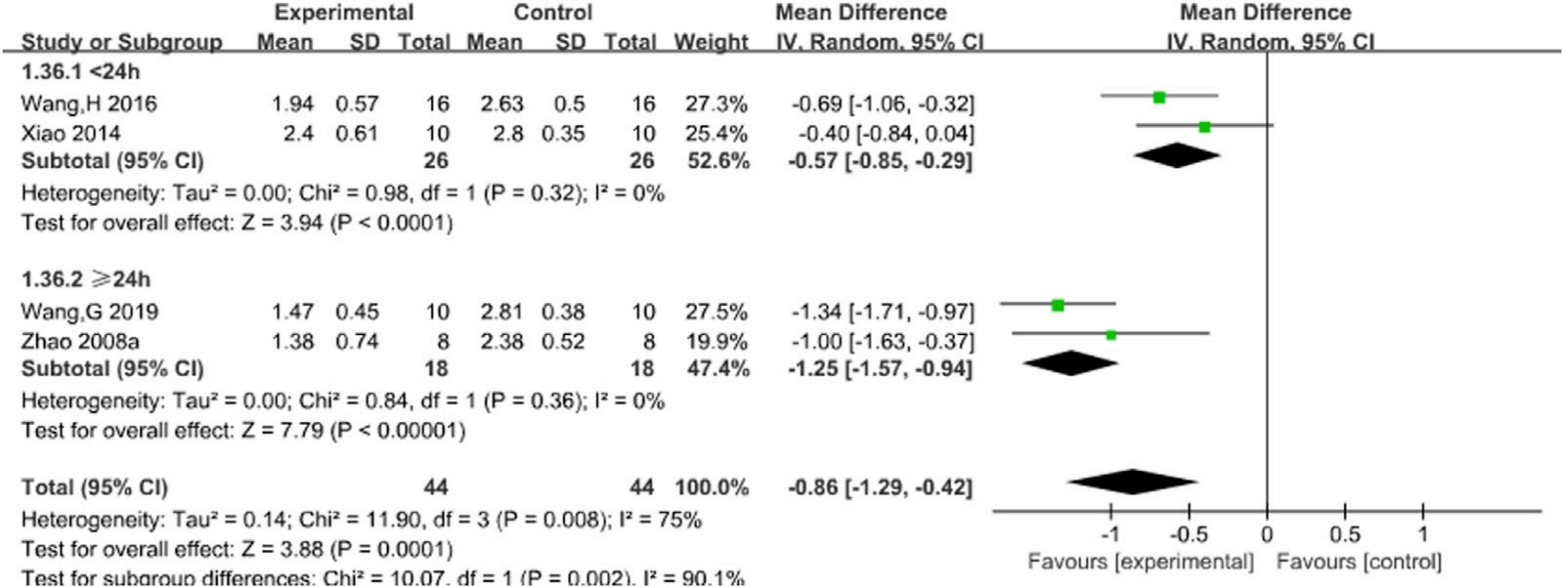
Forest chart of neurological deficit score divided into subgroups according to the treatment time.

Subgroup analyses were conducted using the duration of treatment as the grouping criterion (<24 or ≥24 h). The results showed that Sal B could effectively reduce the neurological deficit score (Figure 4).

#### 3.4.3 Brain index

**FIGURE 5:**
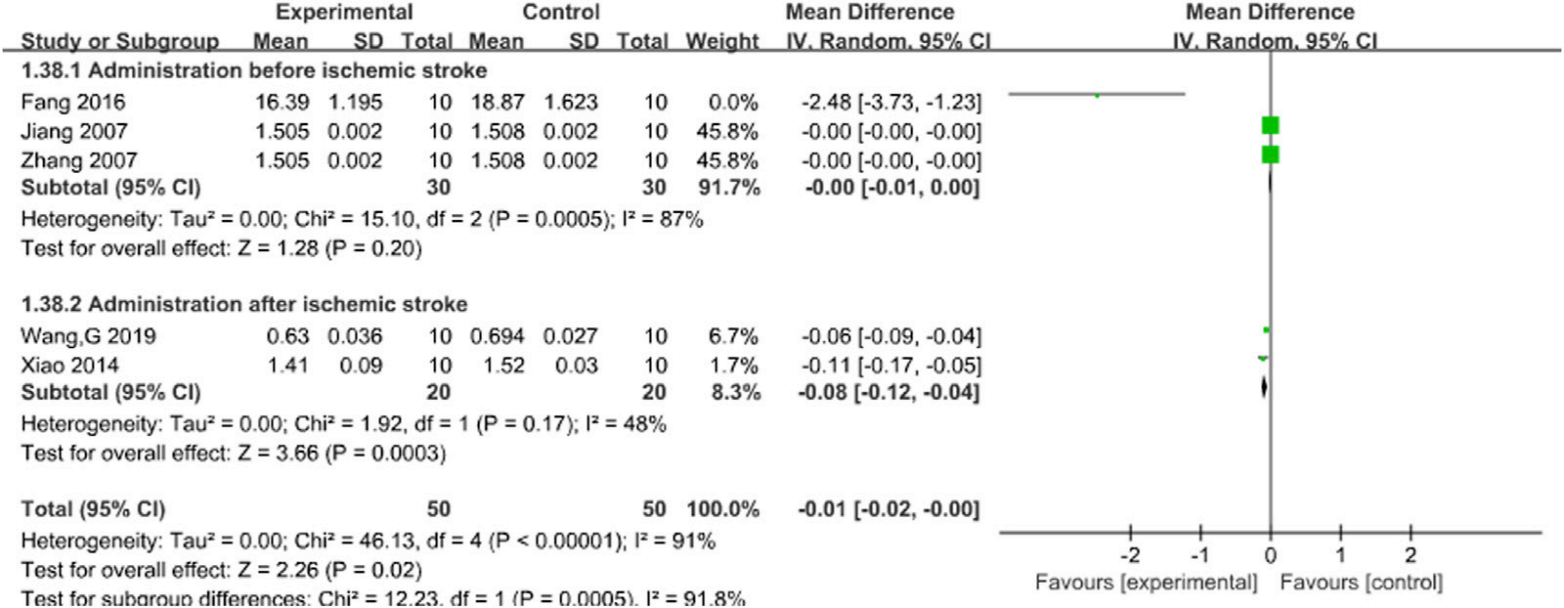
Forest chart of brain index divided into subgroups according to administration time point.

Subgroup analysis was conducted based on the timing of administration. The results showed that there was still a significant difference in the pre-ischemia group, and the difference was not statistically significant (I^2^ = 87%, *P* = 0.20). However, I^2^ decreased significantly to 48% (*P* = 0.0003) in the post-ischemia group, suggesting that timing of administration may be a potential source of brain index heterogeneity. The results support that Sal B can effectively reduce brain index. See Figure 5.

#### 3.4.4 Brain water content

**FIGURE 6:**
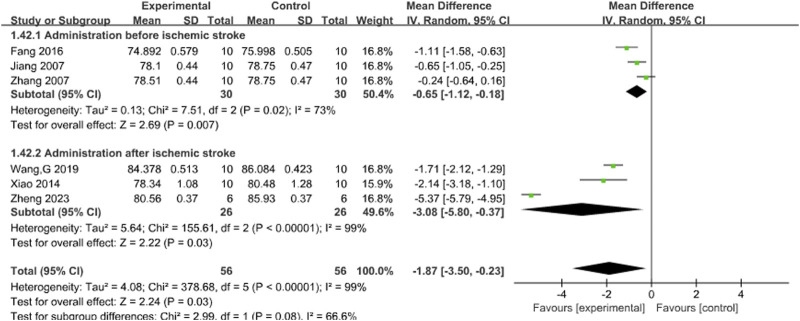
Forest plot of brain water content divided into subgroups according to the timing of administration.

The timing of administration was used as the criterion for subgroup analysis. In subgroup analysis of brain water content, It was observed that I^2^ decreased to 73% (*P* = 0.007) in the pre-ischemia group, whereas I^2^ remained at 99% after ischemia. Thus, different timing of administration may be the primary source of heterogeneity, and the results support the efficacy of Sal B in reducing brain water content after ischemia. See Figure 6.

#### 3.4.5 SOD

**FIGURE 7:**
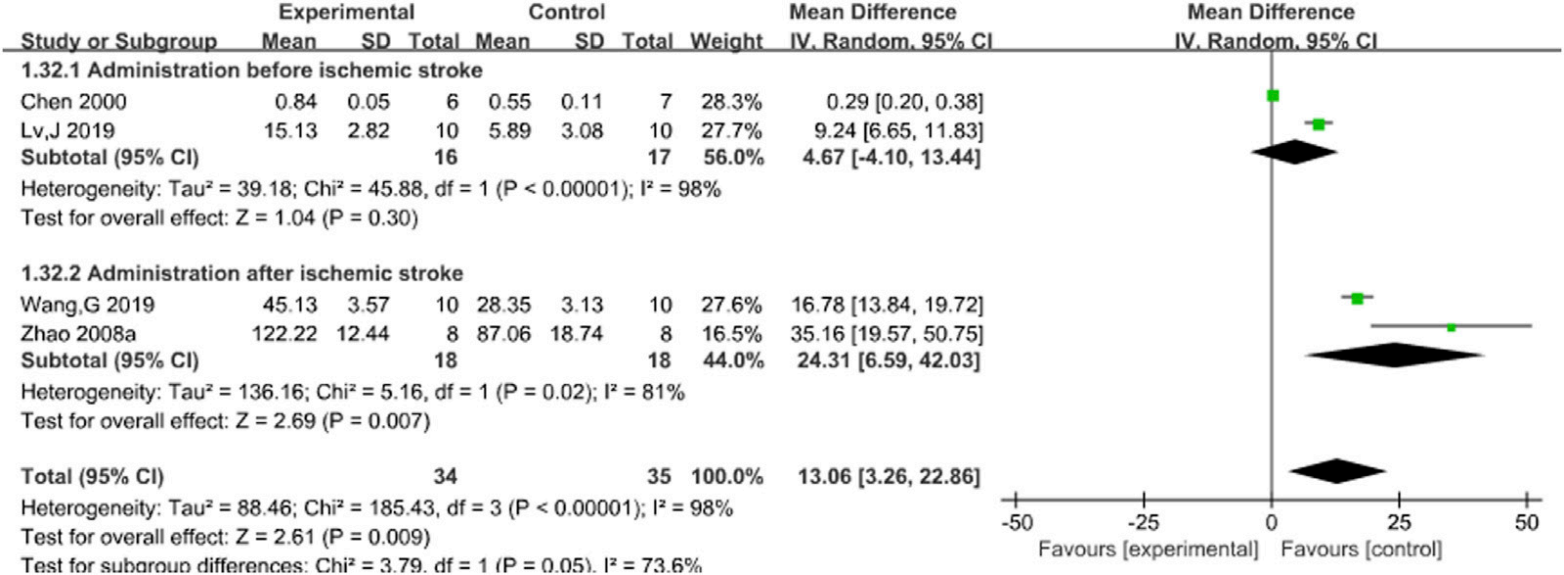
Forest plot of SOD divided into subgroups according to the timing of administration.

Subgroup analysis was conducted based on the timing of administration. Results indicated no statistical significance in administration before acute ischemic stroke (*P* = 0.30), but I^2^ decreased to 81% after acute ischemic stroke (*P* = 0.007), which was statistically significant, suggesting that different timing of administration may be the potential source of heterogeneity. Nevertheless, the results support the ability of Sal B to increase SOD content after acute ischemic stroke. See Figure 7.

#### 3.4.6 MDA

**FIGURE 8:**
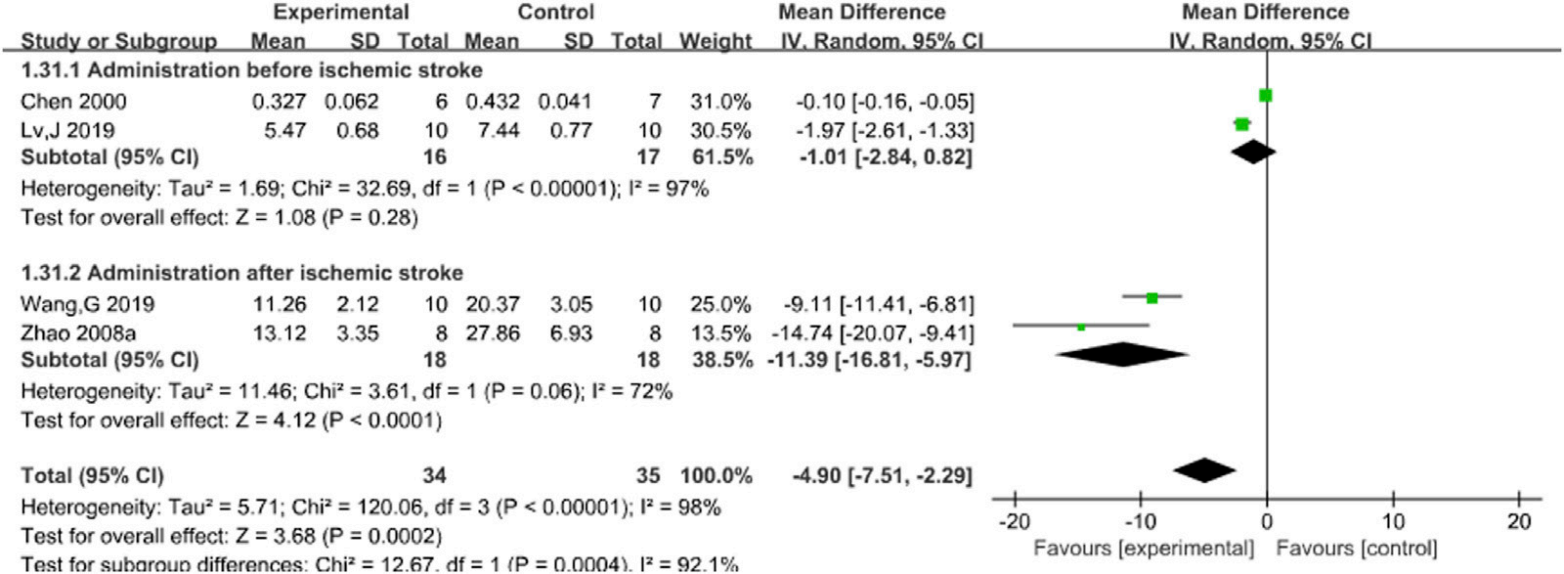
Forest chart of MDA divided into subgroups according to administration time point.

Subgroup analysis was conducted based on the timing of administration. The results showed that there was no statistical significance in preischemic stroke administration (*P* = 0.28), but Z was 4.12 (*P* < 0.00001) after ischemic stroke administration. This finding was statistically significant, suggesting that different timing of administration might be a potential source of heterogeneity. However, the results still support the efficacy of Sal B in reducing MDA content after acute ischemic stroke. See Figure 8.

#### 3.4.7 The number of TUNEL-positive cells

**FIGURE 9:**
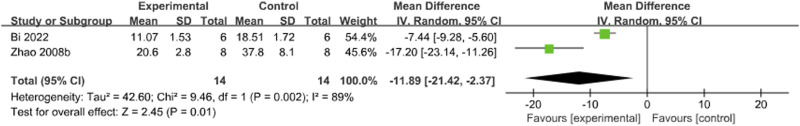
Forest plot for sensitivity analysis of the number of TUNEL-positive cells.

After systematically excluding individual studies, it was found that The study by Lv et al. (2019), may be the primary source of heterogeneity, and I^2^ decreased from 97% to 89% after removing the literatures. Due to the limited number of studies included in this analysis, it is speculated that this study had drug administration before ischemia, while the other two studies were administered after ischemia, which ultimately led to heterogeneity. Nonetheless, the findings continue to support the ability of Sal B to decrease the number of TUNEL-positive cells following acute ischemic stroke. See Figure 9.

### 3.5 Publication bias

**FIGURE 10:**
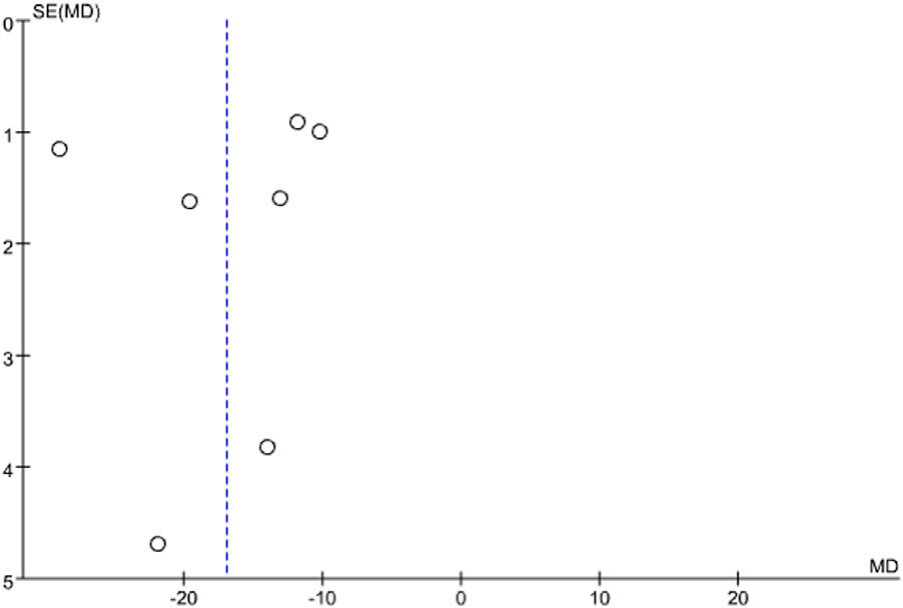
Funnel chart of infarct size.

Publication bias was assessed for infarct size using a funnel plot. The results showed that the points on the funnel plot were asymmetrically distributed along the centerline, indicating the presence of publication bias. See Figure 10.

## 4 Discussion

This meta-analysis demonstrated the effectiveness of Sal B in reducing infarct size and neurological deficit score in animal models of acute ischemic stroke. In addition, the decrease in both brain index and brain water content also reflected that Sal B was effective in alleviating brain edema. This meta-analysis has shown that Sal B can achieve neuroprotective effects through a variety of actions, including:

① Anti-oxidative stress: When the brain tissue is subjected to ischemic injury, a lot of free radicals would be produced and oxidative stress reaction would occur, which results in brain tissue damage. SOD and MDA are common indicators of oxidative stress (Yang et al., 2022; Fu et al., 2022). SOD is a major antioxidant enzyme, which can capture highly active free radicals produced during various chemical reactions and make them inactivate or inert. MDA is the final product of free radicals acting on lipid peroxidation, which has cytotoxicity. Sal B can alleviate brain injury by manipulating the level of SOD and MDA, scavenging oxygen free radicals and inhibiting oxidative stress;
② Anti-inflammatory response: IL-1 is an important pro-inflammatory factor, and IL-1β is the main existing form in brain tissue. Under normal physiological conditions, IL-1β is only expressed at a low level in the brain. After acute ischemic stroke, IL-1β will be released considerably, inducing the production of other inflammatory mediators and resulting in damaging neurons (Yu et al., 2022; Zeng et al., 2022). Sal B can low the level of IL-1β and the expression of inflammatory factors, resulting in reducing the inflammatory response in the brain.
③ Anti-apoptosis: TUNEL staining is often used to evaluate the apoptosis of nerve cells during acute ischemic stroke (Resnick-Silverman, 2021). Sal B could reduce the number of TUNEL-positive cells.
④ Improve energy metabolism: Under aerobic conditions, ADP in the body can react with phosphocreatine (PCr) to generate ATP (Brewster, 2018). Energy charge (EC) is an important parameter that dynamically reflects the state of mutual conversion between ADP and ATP, and a high value of EC indicates active ATP production in cells (Wilson and Matschinsky, 2022). When acute ischemic stroke occurs, aerobic oxidation is impaired, anaerobic glycolysis is enhanced, and the end product lactate is increased and accumulated in tissues, leading to acidosis (Xu et al., 2020). Sal B can increase the EC value and PCr content of ischemic brain tissue, thus inhibiting the production of lactate and improving energy metabolism disorders.
⑤ Stable ion balance: In the early stage of ischemia, energy depletion first affects the function of ion pump. Na^+^/K^+^ ATPase cannot maintain the ion balance in and out of cells when energy is scarce, which leads to the accumulation of sodium in cells (Li et al., 2021; Alquisiras-Burgos et al., 2020). The results showed that Sal B could increase the Na^+^/K^+^ ATPase activity, maintaining ion balance inside and outside the cells.

In addition, we analyzed and summarized other studies that met the criteria for “Sal B in acute ischemic stroke” but could not be combined for analysis. The results showed that Sal B also ameliorated acute ischemic stroke damage by: ① Anti-excitotoxicity: Excessive accumulation of glutamate in the intercellular space is the main source of neurotoxic reactions. The results showed that Sal B can significantly reduce the content of glutamate in brain tissue to inhibit excitatory amino acid toxicity and play a neuroprotective role. ② Anti-platelet aggregation: Platelets are activated and aggregated to form a large number of thrombi to block blood vessels, resulting in insufficient blood and oxygen supply to brain tissue. The results showed that Sal B can effectively combat acute ischemic stroke by inhibiting platelet aggregation and reducing thrombosis. ③ Promoting angiogenesis: VEGF and its receptor molecule VEGFR can improve vascular permeability, promote the generation of cerebral blood vessels by promoting the proliferation and migration of vascular endothelial cells. The results showed that Sal B can promote angiogenesis in time by up-regulating the expression of VEGFR2 and VEGFA, thereby reducing acute ischemic stroke injury.

According to the included studies, 8 studies were administered 7 days to 10 min before cerebral ischemia, and 6 studies were administered immediately or 1 h after cerebral ischemia, both of which had a significant therapeutic effect. The concentration of Sal B between 10 and 192 mg/kg will play a better therapeutic effect, and the longer the treatment time, the more significant the effect. However, more studies are needed to confirm the optimal timing, dosage and duration of administration.

Limitations and future directions: (1) Some studies included in the meta-analysis were downgraded in quality due to lack of blinding during induction and uncontrolled temperature. (2) Given the limited sample size, further research is warranted. (3) It is necessary to further study the mechanism of Sal B. (4) At the same time, better administration timing, the optimal dose and treatment time of Sal B should be understood to improve the efficacy of anti-ischemic stroke.

## 5 Conclusion

In conclusion, this study highlights the significant therapeutic potential of Sal B in treating acute ischemic stroke. Sal B can reduce infarct size, improve neurological deficit symptoms and reduce brain edema by anti-oxidative stress, anti-inflammatory response, anti-apoptosis, improving energy metabolism and stabilizing ion balance, thus achieving neuroprotection. It has great potential in the treatment of acute ischemic stroke.
